# Isolation and Selection of Microalgal Strains from Natural Water Sources in Viet Nam with Potential for Edible Oil Production

**DOI:** 10.3390/md15070194

**Published:** 2017-06-23

**Authors:** Tran Yen Thao, Dinh Thi Nhat Linh, Vo Chi Si, Taylor W. Carter, Russell T. Hill

**Affiliations:** 1 Research Institute for Oil and Oil Plants (IOOP), Ministry of Industry and Trade (MOIT), Ho Chi Minh City 71-175, Vietnam; nhatlinh180490@gmail.com (D.T.N.L); chisi1510@yahoo.com (V.C.S.); 2 Institute of Marine and Environmental Technology (IMET), University of Maryland Center for Environmental Science, Columbus Center Suite 236, 701 East Pratt Street, Baltimore, MD 21202, USA; tcarter357@gmail.com

**Keywords:** microalgal strains, edible oil, saturated fatty acids, unsaturated fatty acids

## Abstract

Industrial vegetable oil production in Viet Nam depends on oil seeds and crude plant oils that are currently more than 90% imported. As the first step in investigating the feasibility of using microalgae to provide Viet Nam with a domestic source of oil for food and edible oil industries, fifty lipid-producing microalgae were isolated and characterized. The microalgae were isolated from water sources ranging from freshwater to brackish and marine waters from a wide geographic distribution in Viet Nam. Initial analyses showed that 20 of the 50 strains had good growth rates, produced high biomass and had high lipid content, ranging up to 50% of dry weight biomass. 18S rRNA gene sequence analyses of the 50 strains showed a great diversity in this assemblage of microalgae, comprising at least 38 species and representatives of 25 genera*: Chlamydomonas*, *Poterioochromonas*, *Scenedesmus*, *Desmodesmus*, *Chlorella*, *Bracteacoccus*, *Monoraphidium*, *Selenastrum*, *Acutodesmus*, *Mychonastes*, *Ankistrodesmus*, *Kirchneriella*, *Raphidocelis*, *Dictyosphaerium*, *Coelastrella*, *Schizochlamydella*, *Oocystidium*, *Nannochloris*, *Auxenochlorella*, *Chlorosarcinopsis*, *Stichococcus*, *Picochlorum*, *Prasinoderma*, *Chlorococcum*, and *Marvania.* Some of the species are closely related to well-known lipid producers such as *Chlorella sorokiniana*, but some other strains are not closely related to the strains found in public sequence databases and likely represent new species. Analysis of oil quality showed that fatty acid profiles of the microalgal strains were very diverse and strain-dependent. Fatty acids in the microalgal oils comprised saturated fatty acids (SFAs), poly-unsaturated fatty acids (PUFAs), and mono-unsaturated fatty acids (MUFAs). The main SFA was palmitic acid. MUFAs and PUFAs were dominated by oleic acid, and linoleic and linolenic acids, respectively. Some strains were especially rich in the essential fatty acid α-linolenic acid (ALA), which comprised more than 20% of the fatty acids in these strains. Other strains had fatty acid compositions similar to that of palm oil. Several strains have been selected on the basis of their suitable fatty acid profiles and high lipid content for further chemical and physical characterization, toxicity and organoleptic tests of their oils, and for scale-up.

## 1. Introduction

Current oil consumption for food and cooking in Viet Nam is 8 to 9 kg/year per capita, which is lower than the 13.5 kg/year per capita world average, and much lower than the 40 kg/year per capita average in the USA and European countries. The World Health Organization recommends the consumption of 20–25 kg/year per capita. In Viet Nam, the demand for edible oils is rapidly increasing, with consumption projections of 16 kg/year per capita in 2020 and 18 kg/year per capita in 2025. Because of the high population in Viet Nam of 92.7 million in 2016, the overall consumption of edible oils in Viet Nam is high and the edible oil industry in Viet Nam depends on oil seeds and crude plant oils for more than 90% of domestic edible oil consumption [1,2], at a cost of several millions of USD.

Microalgae have great potential for oil production, not only for biodiesel but also for edible oils. Microalgae rank among the most efficient biological producers of oil in the plant world, with very high oil yields. Plant oil yields per year per ha have been estimated at 172 L for corn, 446 L for soybean, 2689 L for coconut, and 5950 L for palms, whereas microalgae can produce 136,900 L (assuming 70% oil by weight in the biomass) or 58,700 L (assuming 30% oil) [3]. Microalgae have superior oil production due to their higher photosynthetic efficiency, higher biomass productivity, faster growth rate, and higher CO_2_ fixation rate. Moreover, microalgae can be cultured in areas unsuitable for agriculture and do not require potable water.

Some microalgae produce abundant lipids, and can be induced to produce even higher amounts of lipids by changing growth conditions, generally by starving the microalgae for nitrogen [4,5,6,7,8,9]. Examples of the lipid content of microalgae under normal growth conditions and following nitrogen limitation include *Scenedesmus obliquus*, 11–55%; *Chlorella vulgaris*, 5–58%; *Chlorella sorokiniana*, 19–22%; *Chlorella protothecoides*, 15–58%; and *Nannochloropsis* sp., 12–53% [9]. There are a few studies on microalgae in Viet Nam, particularly for oil production. Reports on the isolation of microalgae in Viet Nam include studies on applications in aquaculture and assessments for biodiesel production [10,11,12].

In this study, we isolated microalgae from natural water sources from many regions in Viet Nam, screened and classified strains having high lipid content as well as potential for high biomass production, and determined fatty acid profiles of selected microalgae. The aim of the research is to investigate the feasibility of using microalgae as sources of edible oils to provide Viet Nam with a domestic source and more economic supply for the food and edible oil industries.

## 2. Results

### 2.1. Isolation of Lipid-Producing Microalgal Strains

**Figure 1:**
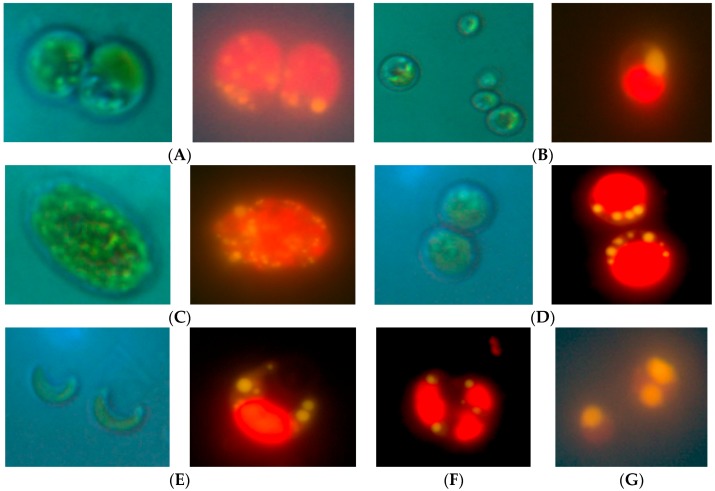
Visualization of lipid droplets in selected microalgal strains by Nile Red staining. (**A**) *Chlorella* sp. N13; left panel, light microscopy; right panel, fluorescence microscopy of Nile Red-stained cells. (**B**) *Nannochloris* sp. M1; left panel, light microscopy; right panel, fluorescence microscopy of Nile Red-stained cells. (**C**) *Chlamydomonas* sp. N1; left panel, light microscopy; right panel, fluorescence microscopy of Nile Red-stained cells. (**D**) *Dictyosphaerium* sp. L2; left panel, light microscopy; right panel, fluorescence microscopy of Nile Red-stained cells. (**E**) *Raphidocelis subcapitata* sp. N18; left panel, light microscopy; right panel, fluorescence microscopy of Nile Red-stained cells. (**F**) *Monoraphidium* sp. L6; fluorescence microscopy of Nile Red-stained cells. (**G**) *Chlorella* sp. L12; fluorescence microscopy of Nile Red-stained cells.

More than 60 microalgae strains naturally existing in the water samples were isolated, obtained in uni-algal culture, and screened by Nile Red staining to qualitatively evaluate lipid production. Fifty strains were selected for further evaluation on the basis of consistent growth and the presence of lipids. Of these 50 strains, 24 were isolated from fresh waters (numbered “N”), 11 strains from brackish waters (numbered “L”), and 15 strains from marine waters (numbered “M”) (Table 1). Microscopic observations at 1000× magnification revealed morphological diversity of the microalgae, and representative morphologies are shown in Figure 1. Lipid droplets were apparent after Nile Red staining, and representative strains containing abundant lipids are shown in Figure 1. 

### 2.2. Molecular Characterization of Microalgal Strains

Analysis of 18S rRNA gene sequences from strains revealed great diversity in this assemblage of microalgae. Based on Basic Local Alignment Search Tool (BLAST) analysis and identity of closest relatives, these strains include representatives of 25 genera: *Chlamydomonas*, *Poterioochromonas*, *Scenedesmus*, *Desmodesmus*, *Chlorella*, *Bracteacoccus*, *Monoraphidium*, *Selenastrum*, *Acutodesmus*, *Mychonastes*, *Ankistrodesmus*, *Kirchneriella*, *Raphidocelis*, *Dictyosphaerium*, *Coelastrella*, *Schizochlamydella*, *Oocystidium*, *Nannochloris*, *Auxenochlorella*, *Chlorosarcinopsis*, *Stichococcus*, *Picochlorum*, *Prasinoderma*, *Chlorococcum*, *Marvania.* Some of the strains are closely related to well-known high lipid producers such as *Chlorella sorokiniana.* Ten of the strains were at 98% or less identity based on 18S rRNA gene sequence comparison to previously characterized strains in GenBank, suggesting that these strains may be representatives of new species or genera. Notably, strains N15 and L6 were 95% and 96% similar to their nearest relatives, indicating that these strains are very likely to be the first cultured representatives of new genera. These strains warrant further taxonomic characterization

In some cases, strains had 100% identity on BLAST analysis to the 18S rRNA gene sequences of reference strains, and these strains can therefore be assigned to species and/or genus with high likelihood. These strains included eight strains isolated from fresh water: *Scenedesmus* sp. N2, *Scenedesmus armatus* N8, *Raphadocelis subcapitata* N18, *Dictyosphaerium* sp. N19, *Scenedesmus* sp. N22, *Scenedesmus* sp. N24, *Chlorella sorokiniana* L1; one strain from brackish water: *Nannochloropsis* sp. L9; and two strains from marine water: *Nannochloris* sp. M2 and *Picochlorum* sp. M5 (Table 1).

### 2.3. Identification of High Lipid-and High Biomass-Producing Microalgae

**Figure 2:**
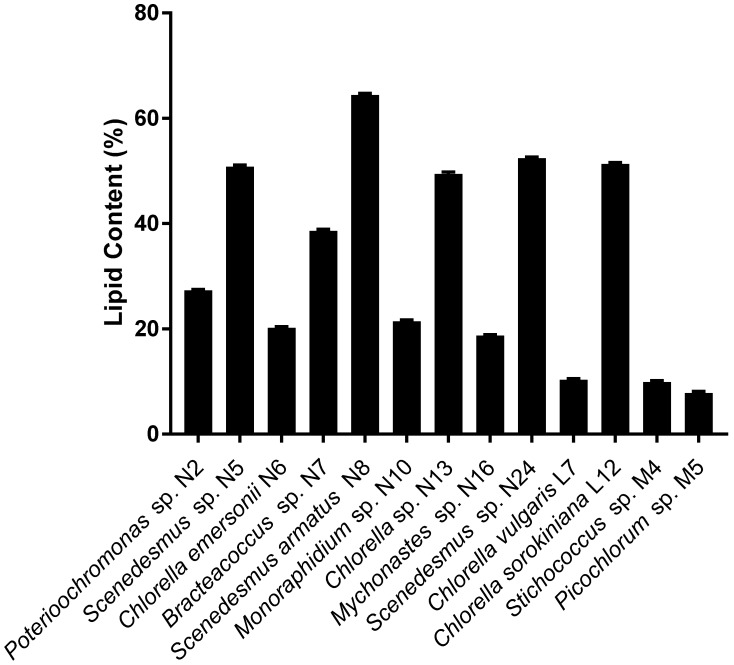
Total lipid as % of dry biomass for 13 selected strains, representing high and low lipid producers in the assemblage of 50 strains.

The lipid content of the strains varied from a low of 7.8% to a high of 64.4%. *Scenedesmus armatus* N8, *Chlorella* sp. N13, *Scenedesmus* sp. N24, and *Chlorella sorokiniana* L12 had lipid contents higher than 50%. The lipid content as a percentage of dry biomass of 13 representative strains of the 50 lipid producers is shown in Figure 2.

**Figure 3:**
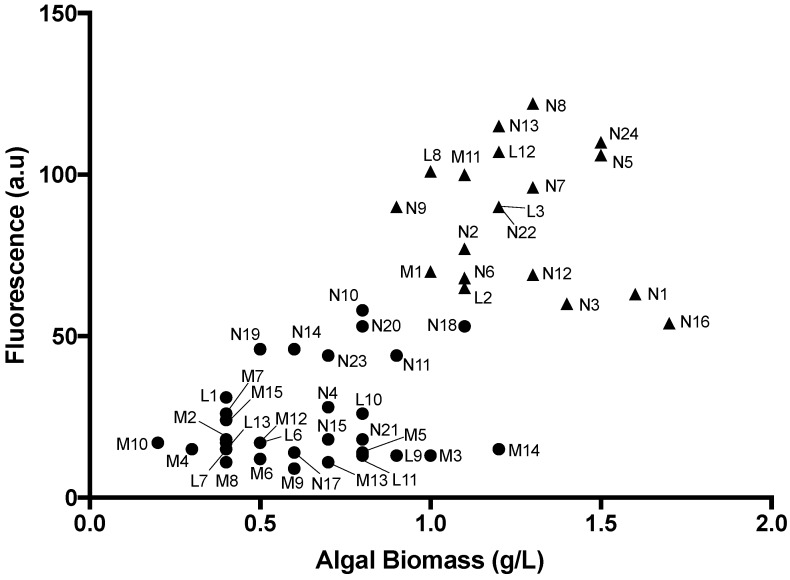
Nile Red fluorescence signal and biomass data for 50 microalgal strains. The 20 strains for which additional lipid characterization was performed are indicated by solid triangles; the remaining strains are indicated by solid circles.

Nile Red fluorescence signal and biomass data are shown in Figure 3. The Nile Red fluorescence intensity of the 50 strains ranged from 9.0 to 122 a.u., and dry biomass ranged from 0.2 to 1.7 g/L. The Nile Red fluorescence intensity of the fresh water microalgae was generally higher than that of microalgae isolated from brackish and marine water. Additionally, biomass production of freshwater strains was generally higher, ranging from 0.5 to 1.7 g/L, whereas it ranged from 0.2 to 1.1 g/L for brackish and marine strains. Based on these data, 20 microalgal strains producing a high amount of lipid as well as high biomass were selected for further analysis: N1, N2, N3, N5, N6, N7, N8, N9, N12, N13, N16, N18, N22, N24, L2, L3, L8, L12, M1, and M11 (indicated by * in Table 1).

### 2.4. Fatty Acid Composition of Microalgal Lipids

Table 2 and Table 3 show fatty acid profiles of the 20 promising strains listed above. Fatty acids of the strains were from C8 to C24. There was a range in complexity of the fatty acid profiles in the various strains. Strains N18, N22, and L3 had 17 different fatty acids in their profile, whereas strains N9 and L8 had 12 fatty acids, and M1 and M11 had only seven and six fatty acids, respectively.

Saturated fatty acids varied significantly between strains from a low of 22.3% (*Scenedesmus* sp. N5) to a high of 78.1% (*Mychonastes* sp. N16). Palmitic acid (C16:0) was dominant in the saturated fatty acids, from 46.9 to 93% of total saturated fatty acids. Five of the 20 strains had more than 80% palmitic acid in their total saturated fatty acids. The second major saturated fatty acid was steric acid (C18:0), ranging from 4.8% (*Nannochloris* sp. M1) to 18.9% (*Poterioochromonas* sp. N2). Other fatty acids included caprylic C8:0, lauric C12:0, myristic C14:0, margaric C17:0, arachidic C20:0, behenic C22:0 and lignoceric C24:0 acids. 

The major unsaturated fatty acids in the 20 strains were oleic (C18:1n-9), linoleic (C18:2n-6), and linolenic (C18:3n-3) acids, followed by palmitoleic acid (C16:1). Unsaturated fatty acids with one double bond (mono-unsaturated fatty acids, MUFAs) ranged from 7% (*Picochlorum oculatum* M11) to 35.4% (*Chlorella* sp. N13), and there were seven strains having more than 30% MUFA. Poly-unsaturated fatty acids (PUFA) contents ranged from 3% (*Mychonastes* sp. N16) up to 39.8% (*Oocystidium* sp. L8) in the total fatty acids. The PUFA content of seven strains was more than 20%, while another seven strains had less than 20%. In the unsaturated fatty acids, oleic acid (C18:1) predominated, ranging from 8.3% (*Nannochloris* sp. M10) to 38.5% (*Chlorella sorokiniana* L12). α-linolenic acid (ALA)—an omega 3 essential fatty acid—was much higher in some strains than others, at 22.4%, 20.4%, 18.5%, and 17.2% in *Scenedesmus* sp. N5, *Scenedesmus* sp. N24, *Oocystidium* sp. L8, and *Picochlorum oculatum* M11, respectively.

## 3. Discussion

The isolation of microalgae that produce a high lipid content and high biomass is a prerequisite for the successful industrial production of edible oils. In order to isolate good microalgal candidates from nature, 86 water samples were taken from a broad range of geographic sites in 15 provinces of Viet Nam lying between latitudes 9° to 21° N and longitudes 104° to 109° E. In the North, the location is latitudes 20°54′ to 20°57′ and longitudes 106°59′to 107°01′. Water samples were collected from a great diversity of aquatic habitats, including freshwater rivers and lakes, brackish mangroves and melaleuca forests, and marine samples from coral reefs, seagrass beds, and open ocean.

Strains were identified by sequencing of 18S rRNA gene fragments and BLAST analysis, revealing that a great diversity of strains was successfully isolated, with at least 38 different species belonging to 25 genera. We attribute this diversity to the broad geographic range of sampling from highly diverse environments. The salinity of water samples ranged from 1 to 36 psu. Salinity is a key physical parameter that affects plankton diversity [13,14]. Other important factors that affect microalgal diversity are temperature and nutrient availability [15,16]; these two factors also varied widely in the water samples collected in this study. 

Biomass production and lipid content varied widely in our strains. The two highest biomass-producing strains (N1 and N16) contained moderate amounts of lipid. In general, microalgal strains do not produce high lipid content under conditions of rapid growth [3,6,17,18,19,20,21,22]. Many of our strains that produced moderate to high amounts of lipid (above 60 fluorescence units in Figure 3) also produced high biomass yields of ca. 1 g/L or higher. We attribute this to the fact that strains were grown for 15–20 days and may have sufficiently depleted the nitrogen in the media to induce high lipid production. It is important to note that cultures were bubbled with air rather than CO_2_. The use of air makes the scale-up process more simple and economical. However, the use of CO_2_ may improve growth and provide greater biomass. Further optimization of growth and lipid production is likely still achievable in these strains. 

Total lipid content measured by a chemical method in 13 representative strains (Figure 2) confirmed the five strong candidates for large-scale oil production (i.e., high oil content as well as high biomass production, shown in Figure 3). These strains are *Scenedesmus armatus* N8, *Chlorella* sp. N13, *Scenedesmus* sp. N24, and *Chlorella sorokiniana* L12, which had lipid contents higher than 50%, and *Scenedesmus* sp. N5 with a lipid content of almost 50%. There was a good concordance between lipid content measured chemically and as assessed by Nile Red staining in these five highest lipid-containing strains.

Lipids are essential nutrients for humans. For the commercial production of edible oils from microalgae, the candidates for oil production must have not only high oil content and high biomass, but also suitable fatty acid composition, because this affects the quality of the oil and suitability for human consumption. The fatty acid composition of oils from 20 promising strains was determined and found to be very diverse and strain-dependent. Similar to vegetable oils, the fatty acids in oils of microalgae are saturated fatty acids (SFAs) and unsaturated acids (UFAs). Unsaturated acids consist of monounsaturated fatty acids (MUFAs) and polyunsaturated fatty acids (PUFAs). The main SFA is palmitic acid. The main MUFAs oleic acid and linoleic and linolenic acids are dominant in the PUFAs. Three strains have fatty acids comprising more than 75% saturated fatty acids: *Chlamydomonas* sp. N1, *Mychonastes* sp. N16, and *Raphidocelis subcapitata* N18. Unsaturated acids ranged from around 20% (*Raphidocelis subcapitata* N18) to almost 60% (*Scenedesmus* sp. N5), but in most strains they varied from 30 to 50%. Regarding individual fatty acids of saturated or unsaturated composition, there were also variations between the strains. The range in fatty acid profiles produced by the microalgal strains in our assemblage means that the assemblage provides a good starting point for “mixing and matching” oils to obtain various oil compositions that could meet the demands of the edible oil industry.

The fatty acid compositions of our microalgae compare well with those of vegetable oils from conventional oil-producing crops. The saturated and unsaturated fatty acids of palm oil are around 50% each. Soybean and peanut oils are unsaturated oils with around 80% unsaturated fatty acids [23,24]. Palm oil is known as a good cooking oil due to its good oxidative stability and resistance to the formulation of oxidized polymers. These characteristics are likely related to the balance of saturated and unsaturated fatty acids in palm oil. This oil is used in the food industry when the temperature of processing is high, such as deep-frying or baking of food at very high temperature. Some strains of our microalgal assemblage have fatty acid profiles quite similar to palm oil in types and proportions of fatty acids. For example, *Chlorella* sp. N13 has palmitic acid C16:0 (33.2%), oleic acid C18:1 (31.6%), and linoleic acid C18:2 (11.2%) as three main fatty acids. For comparison, the palmitic acid of palm oil is 42.7%, oleic acid 39.3%, and linoleic acid 10.6%. 

The essential fatty acid ALA in the strains *Scenedesmus* sp. N5, *Scenedesmus* sp. N24, *Oocystidium* sp. L8, and *Picochlorum oculatum* M11 was very high, ranging from about 17% to 22% in the total fatty acids. By comparison, this essential fatty acid ALA is low in conventional vegetable oils, at around 6.7% in soybean oil, 0.23% in peanut oil, 0.3% in sesame oil, and 0.7% in olive oil [25]. These strains could therefore be valuable sources to produce oils rich in ALA.

The edible oil industry of Viet Nam depends on the importation of materials (crude oil and oil seeds) for more than 90% of edible oil production, because the domestic production of oil seed crops does not meet the demand. Viet Nam imports mainly crude palm oil and to a lesser extent soybean and other oil seeds. Production of oil crops in Viet Nam is quite small: around 100,800 Ha of soybean and 200,000 Ha of peanuts, with seed quantities of 146,400 tons and 451,800 tons, respectively, in 2015 [1].

Microalgae could be a good alternative source of plant oils for edible oil production as well as oils for pharmaceutical and cosmetic uses and potentially for biodiesel production, if economically viable production can be achieved from microalgae. Microalgae like plants convert sun energy by photosynthesis to chemical energy, but their growth rates and productivity are much higher compared to oil crops [3]. The fact that we have found microalgal species well suited to local environments that produce high quantities of fatty acids of varied composition is an important first step in achieving edible oil production from microalgae. Additional testing is needed to check for toxicity and to determine organoleptic characteristics of the oils. It is well established that lipid content and biomass productivities as well as the quality of microalgae oil can be improved by appropriate growth conditions and by induction to accumulate substantial quantities of lipids [25,26,27,28,29]. In on-going work, parameters such as temperature, pH, salinity, CO_2_, light wavelength and intensity, and nutrient concentrations are being tested to optimize oil production from our most promising strains. The ultimate aim is to set up an oil production process for edible oil production. The vegetable oil industry of Viet Nam is well established and based on advanced technology with modern equipment and appropriate automation. This current industrial system could readily be adapted to include extraction and refining of oil from microalgae.

## 4. Materials and Methods

### 4.1. Water Sampling

Water samples were collected in sterile 50 mL tubes using aseptic techniques from surface waters at depths between 10 and 100 cm below the surface. Eighty-six water samples were collected from lakes, rivers, river mouths, and the sea from many locations in 15 provinces in Viet Nam, including Quang Ninh (Ha Long Bay), Khanh Hoa (Nha Trang, Hon Mun Island, Binh Ba Island), Phu Yen, Binh Thuan, Dac Nong, Ho Chi Minh City (Can Gio beach), Dong Nai, Tiền Giang, Ben Tre, Tra Vinh, Can Tho, Đong Thap, An Giang, Kien Giang, and Ca Mau. Fresh water was collected from two river systems: the Ba and Cuu Long rivers. Water samples were collected from the vicinity of coral reefs at Hon Mun Coral Island (Khanh Hoa Province), from a mangrove forest (Can Gio, Ho Chi Minh City), from a melaleuca forest (Dong Thap province), and from a seagrass sanctuary (Phu Quoc Island, Kien Giang province). The salinity of water samples ranged from 1 to 36 psu and samples were classified into three groups: fresh water (1–2 psu), brackish (3–30 psu), and salt water (>30 psu).

### 4.2. Isolation of Lipid-Producing Microalgae

The media for the isolation of algal strains were BG11 [30] for fresh water, f/2B for brackish, and modified f/2 [31] for marine water samples. Medium f/2B was the same as f/2, except that the seawater was diluted with an equal volume of deionized water. For all three media, solid medium was prepared by the addition of 10 g/L agarose and semi-solid medium by the addition of 5 g/L agarose. Microalgae were isolated directly from the water samples or from enrichment cultures comprising liquid BG11, f/2B, or f/2 that was inoculated with ca. 1/100 inoculum of the water sample and incubated in the light for 10–15 days. Two methods of isolation were applied: streaking on solid agarose plates and culturing in semi-solid agarose plates. For the streaking method, 0.1 mL of the sample (water sample or enrichment culture) was inoculated to the surface of a solid agarose plate. In the semi-solid culturing method, solid medium plates were prepared that contained ca. 15 mL of solid medium. Samples (0.1 mL) were added to 5 mL of semi-solid medium in a small bottle, briefly vortexed to mix, and poured over the solid medium to form a semi-solid overlay. All cultures were incubated at room temperature (30 ± 2 °C), under fluorescent light (12 h light/12 h dark cycles) for 2 to 4 weeks. Isolates were selected on the basis of colony morphology, considering colony color, size, and morphological differences and transferred to fresh agarose plates. The process was repeated as necessary to pure isolates, judged by visual and microscopic examination, until uni-microalgal cultures were obtained. 

### 4.3. Detection of Lipid-Producing Microalgae

Microalgal isolates were assessed for lipid production by using the general approach of Cooksey et al. [32]. A stock of Nile Red (Sigma) was prepared at a concentration of 0.25 mg/mL in ethanol. In initial qualitative screening, isolates were stained with Nile Red by mixing microalgal cultures (96 μL) and 25% (*v*/*v*) DMSO in water (100 μL). Samples were vortexed and held at room temperature for 10 min. The Nile Red stock (4 μL) was added, and samples incubated at 45 °C for 10 min. Cells were then observed for red fluorescent inclusions by fluorescent microscopy to qualitatively assess neutral lipid content. Strains that were observed to have high lipid content were then further assessed. Samples were prepared in the same way and dispensed in microtiter plates together with Nile red standards. The fluorescence intensity was measured in a Synergy HTX plate reader with excitation and emission wavelengths of 530 and 590 nm, respectively.

### 4.4. Sequencing of 18S RNA Genes

DNA was extracted from dense microalgal cultures by using the Qiagen DNeasy Plant Mini Kit. Microalgae culture (200 μL) was pipetted into 900 μL cetyl trimethylammonium bromide CTAB solution. 18S rRNA gene fragments were amplified by using primers EukA-F and EukA-R, Euk-F and Euk-R, and EukB-F and EukB-R; amplicon sizes for primer pairs were 940 bp, 720 bp, and 690 bp, respectively. EukA-F and EukB-R correspond to primers Euk-A and Euk-B [33]. Other primers were designed and modified by Viet A Corp., Ho Chi Minh City, Viet Nam, based on the sequences of the primers of Medlin et al. [33]. Primer sequences are provided in Table S1. Polymerase chain reaction (PCR) reactions were done in 25 μL volumes containing PCR master Mix (12.5 μL-*i*Premium*^i^*^VA^ PCR Master Mix; Viet A Corp.), primers (0.25 μL each of 100 μM), DNA (5 μL; final concentration 100 ng), H_2_O (7 μL). (PCR conditions were one cycle at 95 °C for 5 min; 40 cycles at 94 °C for 30 s, 55 °C for 30 s, and 72 °C for 30 s, and one cycle at 72 °C for 6 min. PCR products were visualized by gel electrophoresis to confirm quality of amplifications and sequenced by the Sanger method using an ABI PRISM^®^ 3730XL Analyzer. Closely related sequences were identified by searching the GenBank nucleotide collection using the Basic Local Alignment Search Tool (BLAST) algorithm at the National Center for Biotechnology Information (NCBI) website. 

### 4.5. Screening of Microalgae for High Biomass Production

All strains were grown in volumes of 150 mL in glass tubes placed under fluorescent lighting (12 h light/12 h dark cycle) of photosynthetically active radiation (PAR) intensity 54 μm m^−2^ s^−1^at 30 °C with air supplied by bubbling at 10 L/h. Cultures were harvested after 15 days for strains isolated from fresh water and after 20 days for strains isolated from brackish and marine water. Biomass was collected by centrifugation at 10,000× *g* for 10 min and dried at 105 ± 2 °C to constant weight.

### 4.6. Lipid Analyses

Total lipid content was determined as described by Bligh and Dyer [34] with minor modifications [35]. Briefly, lipids were extracted by using a mixture of chloroform and methanol (1:2, *v*/*v*). Fatty acids were determined by following the ISO 5509:1994 by GC-ISO/CD 5509:1994 standard method for animal fats and oils preparation of methyl esters of fatty acids followed by gas chromatographic analysis of methyl esters performed using a gas chromatograph (Shimadzu 2010) equipped with a FFAP column and a flame ionization detector.

## 5. Conclusions

This study identified 50 lipid producing strains from many different water sources, ranging from freshwater to brackish and marine water samples in Viet Nam. There were 20 of 50 strains having high lipid and high biomass. Some of the strains had lipid content up to 50% of dry weight biomass. Analysis of 18S rRNA gene sequences of 50 strains showed a great diversity in this assemblage of microalgae. It consists of at least 38 species and representatives of 25 genera: *Chlamydomonas*, *Poterioochromonas*, *Scenedesmus*, *Desmodesmus*, *Chlorella*, *Bracteacoccus*, *Monoraphidium*, *Selenastrum*, *Acutodesmus*, *Mychonastes*, *Ankistrodesmus*, *Kirchneriella*, *Raphidocelis*, *Dictyosphaerium*, *Coelastrella*, *Schizochlamydella*, *Oocystidium*, *Nannochloris*, *Auxenochlorella*, *Chlorosarcinopsis*, *Stichococcus*, *Picochlorum*, *Prasinoderma*, *Chlorococcum*, *Marvania*. Some of the species are closely related to well-known lipid producers like *Chlorella sorokiniana,* but some other strains are not highly related to the strains found in the public genetic database and are possibly novel strains. 

Analysis of the oil quality showing fatty acid profiles of the microalgal strains was very diverse and dependent on strains. Not unlike vegetable oils, fatty acids of the microalgal oils are saturated fatty acids (SFAs) and unsaturated fatty acids (UFAs), including mono-unsaturated fatty acids (MUFA) and poly-unsaturated fatty acids (PUFA). The main SFA is palmitic acid, the main MUFA is oleic acid, and linoleic and linolenic acids are the dominant PUFAs. Some strains are especially rich in the essential fatty ALA, comprising more than 20% of fatty acids in these strains. Some other strains have fatty acid compositions similar to palm oil. Several strains have been selected on the basis of their suitable fatty acid profiles and high lipid content for further chemical and physical characterization, for toxicity and organoleptic tests of their oils, and for scale-up.

## Figures and Tables

**Table 1:** Identification of 50 native strains of microalgae isolated in Viet Nam based on morphology, similarity between amplified sequences, and nearest recorded sequence in the National Center for Biotechnology Information (NCBI) using the Basic Local Alignment Search Tool (BLAST). Twenty microalgal strains producing a high amount of lipid as well as high biomass that were selected for further analysis are indicated with an asterisk.

No.	Name	Species	Nearest Relative	GenBank Number of Relative
1	N1 *	*Chlamydomonas* sp.	99%	FR865562.1
2	N2 *	*Poterioochromonas* sp.	99%	AM981258.1
3	N3 *	*Scenedesmus* sp.	100%	KF569754.1
4	N4	*Desmodesmus communis*	97%	KF864475.1
5	N5 *	*Scenedesmus* sp.	99%	FR865732.1
6	N6 *	*Chlorella emersonii*	99%	FR865661.1
7	N7 *	*Bracteacoccus* sp.	97%	JQ259919.1
8	N8 *	*Scenedesmus armatus*	100%	KR082490.1
9	N9 *	*Chlorella sorokiniana*	99%	KF444207.1
10	N10	*Monoraphidium* sp.	100%	KR061995.1
11	N11	*Selenastrum* sp.	99%	JQ360530.1
12	N12 *	*Acutodesmus* sp.	99%	KT267272.1
13	N13 *	*Chlorella* sp.	99%	FR865687.1
14	N14	*Chlorella* sp.	99%	KT452082.1
15	N15	*Ankistrodesmus gracilis*	94%	KF574394.1
16	N16 *	*Mychonastes* sp.	99%	JN617908.1
17	N17	*Kirchneriella dianae*	99%	HM483512.1
18	N18 *	*Raphidocelis subcapitata*	100%	KF673369.1
19	N19	*Dictyosphaerium* sp.	100%	GQ176860.1
20	N20	*Desmodesmus abundans*	99%	AB917136.1
21	N21	*Muriella decolor*	99%	JN968587.1
22	N22 *	*Scenedesmus* sp.	100%	KF569755.1
23	N23	*Neodesmus* sp.	99%	KJ173793.1
24	N24 *	*Scenedesmus* sp.	100%	KT267272.1
25	L1	*Chlorella sorokiniana*	100%	KR092112.1
26	L2 *	*Dictyosphaerium* sp.	99%	GQ487254.1
27	L3 *	*Coelastrella* sp.	99%	JX513883.1
28	L6	*Schizochlamydella capsulata*	95%	AY044652.1
29	L7	*Chlorella vulgaris*	99%	FR865683.1
30	L8 *	*Oocystidium* sp.	96%	HQ008711.1
31	L9	*Nannochloris* sp.	100%	JQ315642.1
32	L10	*Auxenochlorella protothecoides*	98%	KM462820.1
33	L11	*Chlorella* sp.	99%	KP262476.1
34	L12 *	*Chlorella sorokiniana*	99%	KF864476.1
35	L13	*Mychonastes afer*	99%	GQ477049.1
36	M1 *	*Nannochloris* sp.	98%	KF791551.1
37	M2	*Nannochloris* sp.	100%	JQ315641.1
38	M3	*Chlorosarcinopsis* sp.	98%	JN76.10864
39	M4	*Stichococcus* sp.	99%	KM020184.1
40	M5	*Picochlorum* sp.	100%	JN191236.1
41	M6	*Chlorella* sp.	99%	EU282452.1
42	M7	*Prasinoderma* sp.	99%	AB183584.1
43	M8	*Picochlorum eukaryotum*	99%	X06425.1
44	M9	*Chlorococcum* sp.	97%	AB183580.1
45	M10	*Picochlorum maculatum*	99%	KM055115.1
46	M11 *	*Picochlorum oculatum*	99%	AY422075.1
47	M12	*Marvania* sp.	98%	KF144207.1
48	M13	*Chlorella minutissima*	99%	HQ218939.1
49	M14	*Picochlorum atomus*	99%	FJ536747.1
50	M15	*Nannochloris* sp.	99%	AB058309.1

**Table 2:** Saturated fatty acids in 20 high lipid-containing microalgal strains isolated in Viet Nam.

Strains	Saturated Fatty Acids (%)											
	C8:0	C10:0	C12:0	C14:0	C15:0	C16:0	C17:0	C18:0	C20:0	C22:0	C24:0	Total
*Chlamydomonas*sp. N1	1.2	0.95	8.56	5.38	0.87	35.66	1.18	14.10	1.92	6.15	-	**75.97**
*Poterioochromonas* sp. N2	-	-	0.38	1.35	-	33.72	0.54	10.56	1.53	3.44	4.27	**55.79**
*Scenedesmus* sp. N3	-	0.17	1.48	1.55	0.24	34.09	0.51	8.57	1.07	3.84	1.43	**52.95**
*Scenedesmus* sp. N5	-	-	0.87	0.70	0.17	18.13	-	1.87	0.09	0.24	0.22	**22.29**
*Chlorella emersonii* N6	-	0.15	0.55	0.63	0.30	44.03	0.68	6.07	0.69	1.74	0.89	**55.73**
*Bracteacoccus* sp. N7	-	-	0.47	2.21	0.37	25.91	-	7.83	1.66	4.25	4.05	**46.75**
*Scenedesmus armatus* N8	1.88	-	1.99	1.79	-	34.72	-	15.00	1.17	4.77	-	**61.32**
*Chlorella sorokiniana* N9	-	-	-	1.69	-	41.81	1.00	7.77	0.88	4.01	4.58	**61.74**
*Acutodesmus* sp. N12	-	-	-	-	-	36.79	1.23	10.33	1.85	5.74	2.29	**58.23**
*Chlorella* sp. N13	-	-	-	0.22	0.06	33.23	0.43	6.22	0.37	0.13	-	**40.66**
*Mychonastes* sp. N16	-	0.30	3.14	2.27	0.56	51.54	0.91	8.97	1.17	2.87	6.56	**78.05**
*Raphidocelis subcapitata* N18	2.29	1.92	5.07	3.34	0.75	40.54	0.95	12.93	1.31	3.93	3.26	**76.29**
*Scenedesmus* sp. N22	0.38	0.47	2.00	3.06	0.47	47.85	0.74	10.38	0.77	2.23	1.04	**69.39**
*Scenedesmus* sp. N24	-	-	-	0.66	0.24	25.22	-	2.19	-	-	-	**28.31**
*Dictyosphaerium* sp. L2	0.55	0.48	3.88	2.76	-	31.46	-	5.09	0.53	1.74	-	**46.49**
*Coelastrella*s p. L3	0.43	0.25	2.82	2.46	0.60	35.98	0.83	14.54	1.26	3.80	-	**62.97**
*Oocystidium* sp. L8	-	-	-	0.97	-	18.53	-	3.60	0.31	2.39	-	**25.80**
*Chlorella sorokiniana* L12	-	-	-	0.35	-	23.48	0.37	5.19	0.52	0.22	-	**30.07**
*Nannochloris* sp. M1	-	-	-	0.63	-	27.08	-	1.41	-	-	-	**29.12**
*Picochlorum oculatum* M11	-	-	-	-	-	29.60	-	2.35	-	-	-	**31.95**

**Table 3:** Unsaturated fatty acids in 20 high lipid-containing microalgal strains isolated in Viet Nam. MUFA: mono-unsaturated fatty acid; PUFA: poly-unsaturated fatty acid.

Strains	Unsaturated Fatty Acid (%)											
	C14:1	C15:1	C16:1	C18:1	C18:2	C18:3 (α )	C18:3 (γ)	C20:1	C22:1	MUFA	PUFA	Total
*Chlamydomonas* sp. N1	-	-	1.83	14.12	2.86	2.59	-	0.56	-	**16.51**	**5.45**	**21.96**
*Poterioochromonas* sp. N2	-	-	2.42	28.45	6.07	1.74	-	1.02	0.71	**32.60**	**7.81**	**40.41**
*Scenedesmus* sp. N3	-	-	-	26.16	8.08	4.28	-	1.23	0.50	**27.89**	**15.38**	**43.27**
*Scenedesmus* sp. N5	0.20	-	7.62	13.51	15.23	22.39	--	0.15	0.20	**21.68**	**38.30**	**59.98**
*Chlorella emersonii* N6	-	-	5.72	26.00	4.39	1.73	-	0.60	-	**32.32**	**6.12**	**38.44**
*Bracteacoccus* sp. N7	-	-	2.59	26.51	11.14	3.25	-	1.21	0.75	**31.06**	**14.39**	**45.45**
*Scenedesmus armatus* N8	-	-	-	30.72	6.29	1.07	-	0.59	-	**31.31**	**7.36**	**38.67**
*Chlorella sorokiniana* N9	-	-	3.02	19.04	7.16	2.43	-	0.29	-	**22.35**	**9.59**	**31.94**
*Acutodesmus* sp. N12	-	-	0.67	29.42	3.67	1.17	-	-	-	**30.09**	**9.43**	**39.52**
*Chlorella* sp. N13	0.09	-	2.78	31.55	11.16	4.22	1.99	0.91	0.07	**35.40**	**17.37**	**52.77**
*Mychonastes* sp. N16	-	-	1.61	14.59	2.24	0.74	-	1.17	0.78	**18.15**	**2.98**	**21.13**
*Raphidocelis subcapitata* N18	-	-	1.53	11.65	3.37	2.31	-	0.81	0.84	**14.83**	**5.68**	**20.51**
*Scenedesmus* sp. N22	-	-	0.54	15.48	5.85	4.14	-	0.92	0.45	**17.39**	**9.99**	**27.38**
*Scenedesmus* sp. N24	-	-	2.11	13.82	8.70	20.36	0.41	-	-	**15.93**	**35.47**	**51.40**
*Dictyosphaerium* sp. L2	-	-	-	8.24	17.08	13.37	-	0.47	0.30	**9.98**	**30.45**	**40.43**
*Coelastrella* sp. L3	-	-	2.64	19.53	6.24	3.82	0.32	1.21	1.14	**24.52**	**10.38**	**34.90**
*Oocystidium* sp. L8	-	-	0.59	6.83	17.22	18.52	0.56	0.13	-	**7.55**	**39.82**	**47.37**
*Chlorella sorokiniana* L12	-	-	0.96	33.80	18.36	4.48	0.05	0.16	-	**34.92**	**22.89**	**57.81**
*Nannochloris* sp. M1	-	-	1.44	7.30	20.04	10.91	-	-	-	**8.74**	**30.95**	**39.69**
*Picochlorum oculatum* M11	-	-	0.51	6.50	22.29	17.20	-	-	-	**7.01**	**39.49**	**46.50**

## Supplementary Materials

The following are available online at www.mdpi.com/1660-3397/15/7/194/s1, Table S1. Sequences of primers used for amplification of 18S rRNA gene fragments from microalgae.
