# Supplementary material for: Isolation and Selection of Microalgal Strains from Natural Water Sources in Viet Nam with Potential for Edible Oil Production
Source: Mar Drugs. 2017 Jun 23;15(7):194. doi: 10.3390/md15070194 (PMC5532636; doi:10.3390/md15070194)
Supplement: Supplementary file 1 [file marinedrugs-15-00194-s001.pdf]

Table S1. Sequences of primers used for amplification of 18S rRNA gene fragments from microalgae.

Primer	Sequence 5′-3′	Reference/source
EukA-F	AACCTGGTTGATCCTGCCAGT	Medlin et al., 1988[1]
EukA-R	CGACGAGCCGAACACGTAC	Designed and modified by Viet A Corp., Ho Chi Minh City, Viet Nam
Euk-F	CACGTACTAGGACTTCGG	Designed and modified by Viet A Corp., Ho Chi Minh City, Viet Nam
Euk-R	GTCAGGAATGGCAGCTTGTGA	Designed and modified by Viet A Corp., Ho Chi Minh City, Viet Nam
EukB-F	CATCGATAGTGTTCGGGA	Designed and modified by Viet A Corp., Ho Chi Minh City, Viet Nam
EukB-R	AAGCTTGATCCTTCTGCAGGTTCC	Medlin et al. 1998[1]

[1]Medlin, L; Elwood, H.J.; Stickel, S.; Sogin, M.L. The characterization of enzymatically amplified eukaryotic 16S-like rRNA-coding regions. *Gene* **1988.** *71*, 491–499.
